# Association between monocyte to high-density lipoprotein cholesterol ratio and kidney stone: insights from NHANES

**DOI:** 10.3389/fendo.2024.1374376

**Published:** 2024-06-04

**Authors:** Zhaoxiang Wang, Guang Zhao, Yuanfei Cao, Tian Gu, Qichao Yang

**Affiliations:** ^1^ Department of Endocrinology, First People’s Hospital of Kunshan, Kunshan, Jiangsu, China; ^2^ Department of Emergency Medicine, First People’s Hospital of Kunshan, Kunshan, Jiangsu, China; ^3^ Department of Urology, First People’s Hospital of Kunshan, Kunshan, Jiangsu, China; ^4^ Department of Endocrinology, Affiliated Wujin Hospital of Jiangsu University, Changzhou, Jiangsu, China; ^5^ Department of Endocrinology, Wujin Clinical College of Xuzhou Medical University, Changzhou, Jiangsu, China

**Keywords:** NHANES, kidney stone, inflammation, oxidative stress, monocyte to high-density lipoprotein cholesterol ratio

## Abstract

**Purpose:**

The ratio of monocyte to high-density lipoprotein cholesterol (MHR) has surfaced as a novel biomarker indicative of inflammation and oxidative stress. The aim of our study was to evaluate the association between MHR and the risk of kidney stones.

**Methods:**

This study analyzed data from individuals aged 20-79 who participated in the National Health and Nutrition Examination Survey (NHANES) between 2007 and 2018. The MHR was assessed as the exposure variable, while a self-reported history of kidney stones was used as the outcome variable. The independent relationship between MHR and the risk of kidney stones was thoroughly evaluated.

**Results:**

This study included 28,878 participants, and as the quartile range of the MHR increased, the proportion of kidney stones also rose progressively (7.20% to 8.89% to 10.88% to 12.05%, *P*<0.001). After adjusting for confounding factors, MHR was independently associated with an increased risk of kidney stones (OR=1.31, 95%CI=1.11-1.54, *P*=0.001), also independent of some common inflammatory indices. Subgroup analysis suggested that the relationship between MHR and kidney stones was more pronounced in female and individuals aged 20-49. Further restricted cubic spline (RCS) analysis indicated a nonlinear relationship between MHR and the risk of kidney stones.

**Conclusion:**

Our results indicate a positive correlation between MHR and an increased risk of kidney stones in US adults, underscoring the need for further large-scale prospective cohort studies to validate these findings.

## 1 Introduction

Kidney stones, a widespread urologic condition, arise from the conglomeration of crystalline minerals and organic molecules within the kidneys or urinary tract (1). The prevalence of the condition has risen significantly, with current global estimates at approximately 10% (2–5). Individuals with kidney stones commonly report a spectrum of discomforts, including pain in the lower back, hematuria, frequent urination, urgency to urinate, and painful urination. If left unaddressed, kidney stones can lead to severe complications, including obstruction of the ureter, infections in the urinary system, and potentially, kidney failure (6). The necessity of surgical procedures for the removal of kidney stones presents a significant economic impact and heightens public health concerns (1).

The development of kidney stones is intertwined with an array of inflammatory responses. Past clinical studies have highlighted some certain inflammatory markers, including the systemic immune-inflammation index (SII) and neutrophil to lymphocyte ratio (NLR), act as predictive biomarkers for the presence of kidney stones (7–9). Monocytes play a pivotal role in the innate immune response, orchestrating the elevation of pro-inflammatory cytokines (10). Concurrently, high-density lipoprotein cholesterol (HDL-c, mmol/L) is recognized for its antioxidative and anti-inflammatory properties (11–13). Previous studies demonstrated HDL-c has the capacity to mitigate and counteract monocyte activation via the inhibition of CD11b, mediated by apolipoprotein A-I (apoA-I) (14). The ratio of monocytes to HDL-c (MHR) has emerged as a potential novel indicator of the balance between the inflammatory and oxidative stress (15).

To date, there is a lack of research examining the link between MHR and the risk of kidney stones. This study, drawing on data from the National Health and Nutrition Examination Survey (NHANES), aims to elegantly dissect the potential association between MHR and the kidney stone risk through a comprehensive cross-sectional analysis.

## 2 Materials and methods

### 2.1 Data source

This population-based study drew upon data from the NHANES, a comprehensive survey conducted by the National Center for Health Statistics of the Centers for Disease Control and Prevention. NHANES employed a rigorous randomized, stratified, multi-stage survey methodology to ensure nationwide representation. Participants underwent thorough physical examinations, health, and nutrition questionnaires, as well as laboratory assessments (16, 17). The NHANES study protocol received approval from the Ethics Review Board of the National Center for Health Statistics. Detailed design and data from this study could be accessed at https://www.cdc.gov/nchs/nhanes/. The current study included a total of 28,878 eligible participants, obtained by consolidating data from the NHANES cycles: 2007–2008, 2009–2010, 2011–2012, 2013-2014, 2015-2016, and 2017-2018, encompassing 59,842 participants. All participants were aged between 20 and 79 years, were not pregnant, had complete data of MHR, and provided comprehensive questionnaire records on kidney stone.

### 2.2 Exposure and outcome definitions

The MHR, serving as an exposure variable, is defined as the quotient of monocyte count to HDL-c levels, with units in 10^9/L and mmol/L, respectively. Three typical indices associated with inflammatory response were also used to represent the effect of inflammation on kidney stones (7, 8, 18). The SII, NLR, and platelet to lymphocyte ratio (PLR) were calculated using the following formulas: SII = platelet x neutrophil/lymphocyte (10^9/L), NLR = neutrophil/lymphocyte (10^9/L), and PLR = platelet/lymphocyte (10^9/L). The assessment of the history of kidney stones, which served as the outcome variable, was determined by asking the question, “Have you or the sample person (SP) ever had a kidney stone?” (ID: KIQ026). Individuals who responded “yes” were categorized as having kidney stones, while those who responded “no” were classified as not having kidney stones. The reliability of self-reported kidney stone history has been established in previous studies (3, 4, 7, 19–21).

### 2.3 Covariate definitions

Demographic data (age, gender, and race) was obtained, along with various potential covariates such as annual household income, educational level, smoking status, hypertension, diabetes, cardiovascular disease, body mass index (BMI, kg/m^2^), alanine transaminase (ALT, U/L), aspartate transaminase (AST, U/L), gamma-glutamyl transferase (GGT, U/L), glycohemoglobin, triglycerides (TG, mmol/L), total cholesterol (TC, mmol/L), low-density lipoprotein cholesterol (LDL-c, mmol/L), blood urea nitrogen (BUN, mmol/L), serum creatinine (Scr, μmol/L), and serum uric acid (SUA, μmol/L). BMI is categorized as follows: <25 kg/m^2^ (normal weight), 25-29.9 kg/m^2^ (overweight), ≥30 kg/m^2^ (obesity). Smokers were identified as current or former smokers. Additionally, self-reported diabetes, hypertension, and cardiovascular disease were recorded. The presence of cardiovascular disease was determined based on self-reported history of heart attack, stroke, congestive heart failure, coronary artery disease, or angina. Comprehensive measurement procedures for all variables were publicly accessible in the NHANES database.

### 2.4 Statistical analysis

The statistical analyses adhered to the Centers for Disease Control and Prevention guidelines, using a complex multistage cluster survey design and weights from six cycles. Continuous variables were presented as means with standard errors (SE), and categorical variables as percentages. The weighted Student’s t-test and chi-squared test compared continuous and categorical variables across groups, respectively. Weighted logistic regression models were used to investigate the associations between monocytes, HDL-c, and MHR (both continuous and quartile) with the risk of kidney stones. Three common models were used: Model 1 was unadjusted; Model 2 adjusted for age, gender, and race; and Model 3 additionally for annual household income, education level, smokers, hypertension, diabetes, cardiovascular disease, BMI, ALT, AST, GGT, glycohemoglobin, TG, BUN, Scr, and SUA. Furthermore, the impacts of SII, NLR, and PLR have been additionally adjusted based on Model 3. Decision curve analysis (DCA) was employed to evaluate the performance of MHR, SII, NLR, and PLR on kidney stone risk. Subgroup analysis was based on age, gender, race, BMI, hypertension, diabetes, and cardiovascular disease stratification. Finally, Restricted Cubic Spline (RCS) analysis further investigated the relationship between MHR and kidney stone risk. For observed non-linear correlations, a two-piecewise linear regression model was used to define intervals and identify threshold effects. All statistical analyses in this study were performed based on the Empower software (http://www.empowerstats.com) and R software (http://www.R-project.org). A *P* value < 0.05 was deemed statistically significant.

## 3 Results

### 3.1 Baseline characteristics of study population

The study included 28,878 participants aged 20 to 79 years, who were not pregnant, had complete MHR data, and provided comprehensive kidney stone questionnaire records. Among them, 2,663 individuals were diagnosed with kidney stones. The average age was 46.25 years, and males constituted 49.21% of the cohort. **Table 1** delineates the comparative analysis of general characteristics and clinical indicators between those with and without kidney stones. The kidney stone group had higher average age, male ratio, BMI, and prevalence of smokers, hypertension, diabetes, and cardiovascular disease. Elevated biochemical levels included glycohemoglobin, GGT, TG, Scr, BUN, and SUA, monocytes, and neutrophils (*P*<0.05). Conversely, this group demonstrated lower levels of HDL-c (*P*<0.001). Significant differences in race distribution were also observed between the groups (*P*<0.001). Crucially, the kidney stone group exhibited higher levels of MHR, SII, and NLR compared to the non-kidney stone group.

**Table 1:** Baseline characteristics of study population in NHANES from 2007 to 2018, weighted.

	Overall (N=28,878)	Non-kidney stone (N=26,215)	Kidney stone (N=2,663)	*P* value
Age (years)	46.25±0.23	45.65±0.24	51.80±0.31	<0.001
Male gender, % (SE)	49.21 (0.32)	48.60 (0.35)	54.85 (1.42)	<0.001
Race, % (SE)				<0.001
Mexican American	8.92 (0.78)	9.20 (0.79)	6.41 (0.76)	
Non-Hispanic Black	10.88 (0.72)	11.42 (0.74)	5.84 (0.56)	
Non-Hispanic White	65.87 (1.43)	64.76 (1.44)	76.13 (1.54)	
Other Hispanic	6.07 (0.50)	6.16 (0.50)	5.28 (0.71)	
Other Races	8.26 (0.46)	8.46 (0.47)	6.34 (0.69)	
Annual household income (under $20,000), % (SE)	13.68 (0.52)	13.66 (0.53)	13.92 (0.87)	0.732
Education level (above high school), % (SE)	61.80 (0.92)	61.81 (0.93)	61.69 (1.58)	0.933
Smokers, % (SE)	44.37 (0.62)	43.81 (0.63)	49.47 (1.51)	<0.001
Hypertension, % (SE)	30.64 (0.52)	29.00 (0.51)	45.72 (1.44)	<0.001
Diabetes, % (SE)	9.50 (0.26)	8.58 (0.27)	17.96 (0.96)	<0.001
Cardiovascular disease, % (SE)	7.47 (0.23)	6.71 (0.24)	14.45 (0.94)	<0.001
BMI (kg/m^2^)	29.17±0.09	28.99±0.09	30.80±0.18	<0.001
ALT (U/L)	25.53±0.14	25.48±0.15	25.93±0.38	0.281
AST (U/L)	25.27±0.11	25.27±0.12	25.24±0.29	0.926
GGT (U/L)	28.32±0.27	28.13±0.29	30.06±0.77	0.022
Glycohemoglobin (%)	5.63±0.01	5.61±0.01	5.84±0.03	<0.001
TG (mmol/L)	1.39±0.02	1.38±0.02	1.52±0.04	<0.001
TC (mmol/L)	5.00±0.01	5.00±0.01	4.98±0.03	0.574
HDL-c (mmol/L)	1.37±0.01	1.38±0.01	1.29±0.01	<0.001
LDL-c (mmol/L)	2.95±0.01	2.95±0.01	2.95±0.03	0.917
BUN (μmol/L)	4.81±0.02	4.77±0.02	5.19±0.06	<0.001
Scr (μmol/L)	77.50±0.27	77.02±0.26	81.88±0.97	<0.001
SUA (μmol/L)	322.37±0.82	321.25±0.85	332.66±2.18	<0.001
Monocytes (10^9/L)	0.56±0.00	0.56±0.00	0.58±0.01	<0.001
Neutrophils (10^9/L)	4.29±0.02	4.27±0.02	4.51±0.05	<0.001
Lymphocytes (10^9/L)	2.15±0.01	2.15±0.01	2.13±0.03	0.330
Platelets (10^9/L)	244.65±0.83	244.92±0.81	242.16±1.82	0.090
MHR	0.45±0.00	0.45±0.00	0.50±0.01	<0.001
SII	530.09±3.53	527.02±3.60	558.39±9.34	0.001
NLR	2.16±0.01	2.15±0.01	2.30±0.30	<0.001
PLR	123.99±0.58	124.05±0.59	123.47±1.32	0.667

Values for categorical variables are given as weighted percentage (standard error); for continuous variables, as weighted mean ± standard error. Weighted Student’s t-test and chi-squared test were used.

BMI, body mass index; ALT, alanine transaminase; AST, aspartate transaminase; GGT, gamma-glutamyl transferase; TG, triglyceride; TC, total cholesterol; HDL-c, high-density lipoprotein cholesterol; LDL-c, low-density lipoprotein cholesterol; BUN, blood urea nitrogen; Scr, serum creatinine; SUA, serum uric acid; MHR, monocyte to high-density lipoprotein cholesterol; SII, systemic immune-inflammation index; NLR, neutrophil to lymphocyte ratio; PLR, platelet to lymphocyte ratio.

### 3.2 Clinical features of the participants according to the quartiles of MHR

Participants were categorized into four quartiles based on their MHR levels (**Table 2**). In comparing the first quartile to quartiles 2-4, there was a notable rise in the percentage of males, annual household incoming under $20,000, smokers, and individuals with hypertension, diabetes, cardiovascular disease, and higher BMI (*P*<0.001). Concurrently, there was a significant increase in biochemical markers such as ALT, AST, GGT, glycohemoglobin, TG, LDL-c, BUN, Scr, SUA, and counts of monocytes, neutrophils, lymphocytes, and platelets (*P*<0.001). Additionally, inflammation indices like SII and NLR also escalated with higher MHR levels (*P*<0.001). In contrast, age, education level above high school, TC, HDL-c, and PLR, were inversely associated with higher MHR levels, and race distribution varied significantly (*P*<0.001). Notably, the prevalence of kidney stones increased progressively from 7.20% in quartile 1 to 12.05% in quartile 4, suggesting a strong association between elevated MHR levels and the risk of kidney stones (*P*<0.001).

**Table 2:** Baseline characteristics of study population according to the quartiles of MHR, weighted.

	Quartile 1 (< 0.29)	Quartile 2 (0.29-0.40)	Quartile 3 (0.40-0.55)	Quartile 4 (> 0.55)	*P* value
Age (year)	47.34±0.33	45.60±0.32	46.10±0.31	46.01±0.32	<0.001
Male gender, % (SE)	27.78 (0.72)	42.44 (0.74)	56.30 (0.72)	69.13 (0.63)	<0.001
Race, % (SE)					<0.001
Mexican American	6.50 (0.56)	9.10 (0.85)	9.91 (0.90)	10.08 (1.00)	
Non-Hispanic Black	14.66 (0.99)	11.99 (0.78)	9.61 (0.69)	7.46 (0.60)	
Non-Hispanic White	63.61 (1.56)	65.42 (1.48)	66.02 (1.53)	68.33 (1.64)	
Other Hispanic	5.22 (0.51)	5.72 (0.52)	6.72 (0.57)	6.57 (0.64)	
Other Races	10.00 (0.66)	7.77 (0.56)	7.75 (0.50)	7.57 (0.51)	
Annual household income (under $20,000), % (SE)	11.79 (0.58)	13.61 (0.67)	14.13 (0.65)	15.12 (0.82)	<0.001
Education level (above high school), % (SE)	69.90 (1.09)	63.15 (1.16)	60.12 (1.06)	54.41 (1.16)	<0.001
Smokers, % (SE)	36.79 (0.96)	40.21 (0.85)	46.65 (1.00)	53.35 (0.86)	<0.001
Hypertension, % (SE)	25.01 (0.75)	27.09 (0.86)	31.93 (0.82)	38.17 (0.88)	<0.001
Diabetes, % (SE)	5.36 (0.32)	7.68 (0.42)	10.38 (0.49)	14.35 (0.53)	<0.001
Cardiovascular disease, % (SE)	4.56 (0.28)	5.49 (0.30)	8.14 (0.37)	11.51 (0.60)	<0.001
BMI (kg/m^2^), % (SE)	26.53±0.12	28.61±0.12	29.90±0.13	31.51±0.11	<0.001
ALT (U/L)	21.64±0.22	23.95±0.28	26.22±0.25	30.08±0.35	<0.001
AST (U/L)	24.57±0.23	24.88±0.24	25.07±0.18	26.52±0.25	<0.001
GGT (U/L)	24.80±0.54	26.69±0.72	28.61±0.39	32.98±0.56	<0.001
Glycohemoglobin (%)	5.47±0.01	5.55±0.01	5.66±0.02	5.84±0.02	<0.001
TG (mmol/L)	1.00±0.01	1.25±0.02	1.48±0.03	2.00±0.04	<0.001
TC (mmol/L)	5.19±0.02	5.02±0.02	4.93±0.02	4.88±0.02	<0.001
HDL-c (mmol/L)	1.80±0.01	1.45±0.01	1.25±0.00	1.03±0.00	<0.001
LDL-c (mmol/L)	2.91±0.02	2.92±0.02	2.97±0.02	3.00±0.03	<0.001
BUN (mmol/L)	4.66±0.04	4.73±0.03	4.88±0.04	4.97±0.04	<0.001
Scr (μmol/L)	73.18±0.47	75.49±0.32	78.92±0.49	82.14±0.40	<0.001
SUA (μmol/L)	290.25±1.26	311.83±1.27	332.54±1.26	353.06±1.43	<0.001
Monocytes (10^9/L)	0.39±0.00	0.50±0.00	0.59±0.00	0.76±0.00	<0.001
Neutrophils (10^9/L)	3.46±0.03	3.99±0.03	4.46±0.03	5.20±0.03	<0.001
Lymphocytes (10^9/L)	1.82±0.01	2.05±0.01	2.22±0.01	2.50±0.01	<0.001
Platelets (10^9/L)	236.08±1.17	241.78±1.00	246.94±1.20	253.31±1.30	<0.001
SII	492.01±6.30	507.30±4.76	540.06±5.82	578.61±5.22	<0.001
NLR	2.08±0.02	2.10±0.02	2.18±0.02	2.28±0.02	<0.001
PLR	140.52±1.00	126.29±0.72	119.56±0.87	110.41±0.73	<0.001
Kidney stone, % (SE)	7.20 (0.44)	8.89 (0.46)	10.88 (0.51)	12.05 (0.60)	<0.001

Values for categorical variables are given as weighted percentage (standard error); for continuous variables, as weighted mean ± standard error. Weighted Student’s t-test and chi-squared test were used.

### 3.3 Associations between the MHR and kidney stone

**Figure 1:**
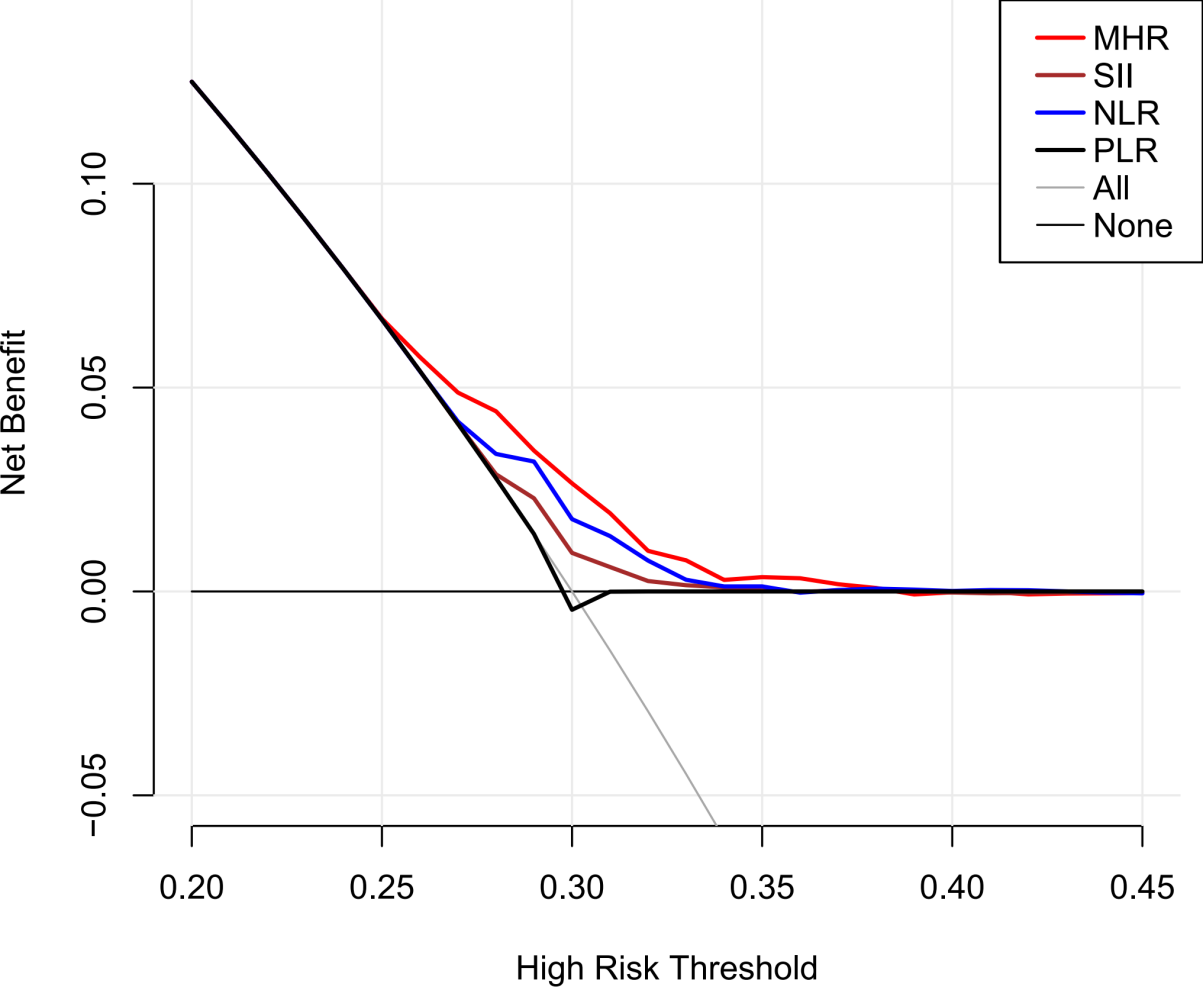
DCA results.

**Table 3** illustrates the relationship between the MHR and kidney stone risk. Initial analyses without adjustment indicated that monocytes and MHR each had a direct correlation with heightened kidney stone risk, whereas HDL-c was inversely correlated (*P*<0.001). These correlations persisted as significant even when adjusted for age, gender, and race (*P*<0.05). Additionally, after fully adjusted for confounding factors, for each unit increase in the MHR, the odds of kidney stone risk rose by 31% (OR=1.31, 95%CI=1.11-1.54, *P*=0.001). It is noteworthy that even after considering the influences of SII, NLR, and PLR, MHR remains an independent risk factor for kidney stones (OR=1.22, 95%CI=1.03-1.44, *P*=0.021). Additionally, according to the results of the DCA analysis, MHR demonstrated superior performance compared to SII, NLR, and PLR (**Figure 1**). When categorizing the MHR into quartiles, the result indicated that individuals in the higher MHR quartiles had a greater prevalence of kidney stones compared to those in the lowest quartile (*P* for trend < 0.001).

**Table 3:** Logistic regression analysis results of MHR and kidney stone.

Kidney stone	OR (95%CI), *P* value			
	Model 1	Model 2	Model 3	Additionally adjusted for SII, NLR, and PLR
Continuous				
Monocytes	1.68 (1.39, 2.03) <0.001	1.24 (1.03, 1.49) 0.025	1.10 (0.90, 1.33) 0.354	0.96 (0.77, 1.20) 0.717
HDL-C	0.57 (0.51, 0.64) <0.001	0.57 (0.51, 0.63) <0.001	0.64 (0.57, 0.72) <0.001	0.65 (0.58, 0.74) <0.001
MHR	1.93 (1.66, 2.25) <0.001	1.61 (1.37, 1.89) <0.001	1.31 (1.11, 1.54) 0.001	1.22 (1.03, 1.44) 0.021
Categories				
Quartile 1	1.00	1.00	1.00	1.00
Quartile 2	1.32 (1.17, 1.49) <0.001	1.30 (1.15, 1.48) <0.001	1.26 (1.11, 1.44) <0.001	1.25 (1.09, 1.42) 0.001
Quartile 3	1.55 (1.37, 1.74) <0.001	1.46 (1.29, 1.65) <0.001	1.36 (1.19, 1.54) <0.001	1.32 (1.16, 1.51) <0.001
Quartile 4	1.79 (1.59, 2.01) <0.001	1.61 (1.43, 1.82) <0.001	1.40 (1.23, 1.60) <0.001	1.35 (1.17, 1.55) <0.001
**P** for trend	<0.001	<0.001	<0.001	<0.001

OR, odds ratio.

95% CI: 95% confidence interval.

Model 2: adjusted for age, gender, and race.

Model 3: adjusted for age, gender, and race, annual household income, education level, smokers, hypertension, diabetes, cardiovascular disease, BMI, ALT, AST, GGT, glycohemoglobin, TG, BUN, Scr, and SUA.

### 3.4 Multivariate logistic regression models of kidney stone

A multivariate logistic regression analysis was performed, with the independent variables including the MHR, SII, NLR, PLR, age, gender, race, annual household income, educational level, smoking, hypertension, diabetes, cardiovascular disease, BMI, ALT, AST, GGT, glycohemoglobin, TG, BUN, Scr, and SUA (**Table 4**). The results showed that MHR, age, male, race, hypertension, diabetes, cardiovascular disease, BMI, and SUA were independent risk factors for kidney stones.

**Table 4:** Multivariate logistic regression models of kidney stone.

	OR	95%CI lower	95%CI upper	*P* value
MHR	1.217	1.030	1.439	0.021
SII	1.000	1.000	1.001	0.159
NLR	0.969	0.885	1.061	0.495
PLR	0.999	0.998	1.001	0.489
Age (years)	1.015	1.010	1.020	<0.001
Gender (vs. female)	1.419	1.226	1.642	<0.001
Race (vs. Mexican American)				
Other Hispanic	0.596	0.467	0.761	<0.001
Non-Hispanic White	1.378	1.140	1.666	0.001
Non-Hispanic Black	1.186	0.932	1.509	0.166
Other Races	1.018	0.789	1.314	0.888
Under $20,000 (vs. no)	1.026	0.880	1.196	0.741
Above high school (vs. no)	1.112	0.976	1.267	0.110
Smokers (vs. no)	0.943	0.829	1.072	0.371
Hypertension (vs. no)	1.231	1.067	1.419	0.004
Diabetes (vs. no)	1.497	1.227	1.826	<0.001
Cardiovascular disease (vs. no)	1.353	1.125	1.626	0.001
BMI (kg/m^2^)	1.032	1.023	1.042	<0.001
ALT (U/L)	1.000	0.994	1.006	0.996
AST (U/L)	0.996	0.989	1.004	0.361
GGT (U/L)	1.000	0.998	1.002	0.733
Glycohemoglobin (%)	0.994	0.932	1.060	0.851
TG (mmol/L)	1.015	0.959	1.074	0.604
BUN (mmol/L)	1.025	0.988	1.062	0.185
Scr (μmol/L)	1.000	0.999	1.002	0.706
SUA (μmol/L)	1.001	1.001	1.002	<0.001

### 3.5 Subgroup analyses

**Figure 2:**
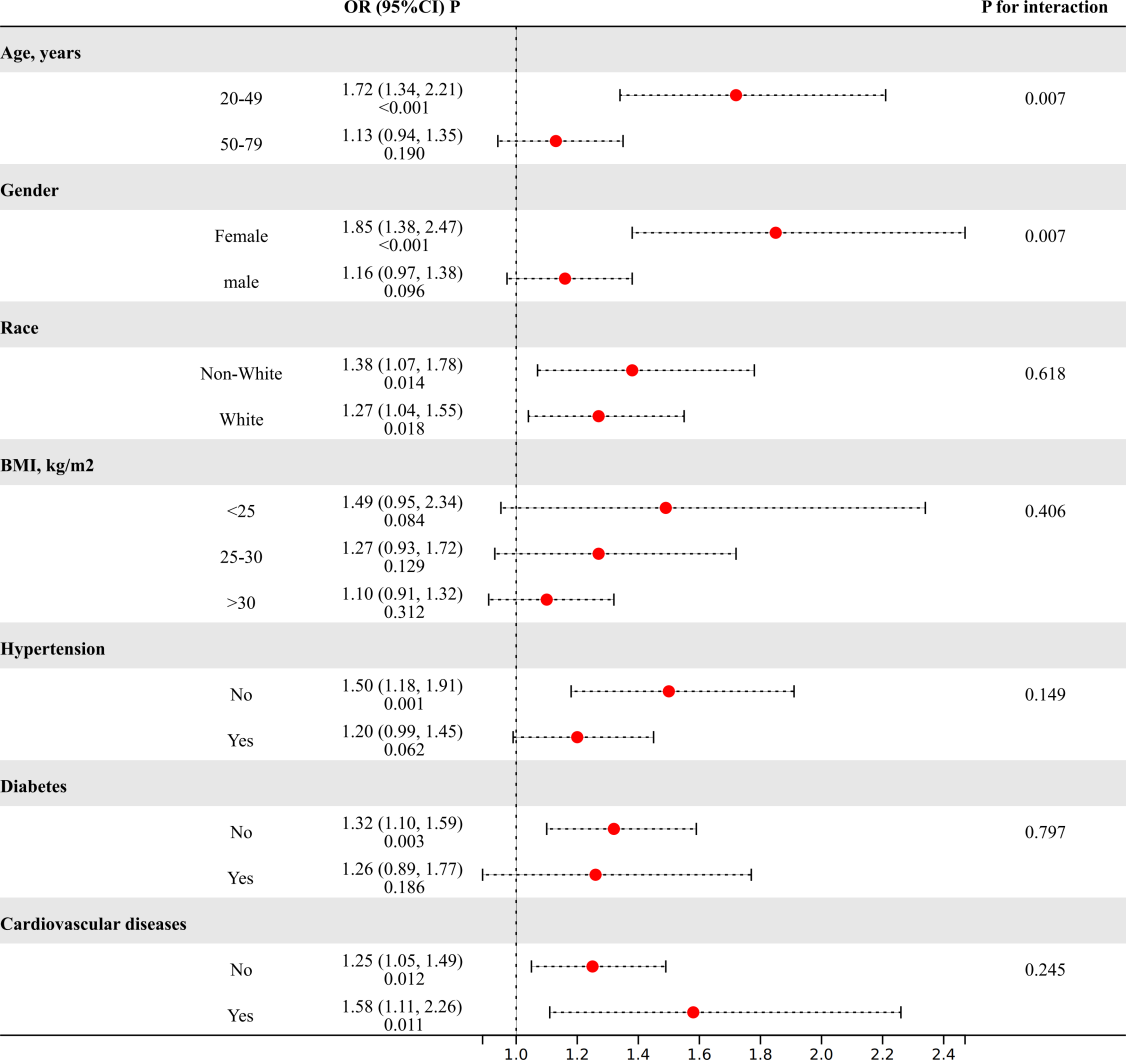
The results of subgroup analyses. (Age, gender, and race, annual household income, education level, smokers, hypertension, diabetes, cardiovascular disease, BMI, ALT, AST, GGT, glycohemoglobin, TG, BUN, Scr, and SUA were adjusted).

The subgroup analysis was performed to evaluate the consistency of the relationship between MHR and the risk of kidney stones across diverse demographic cohorts. The analysis results, depicted in **Figure 2**, demonstrated that stratification by race, BMI, or diabetes, hypertension, and cardiovascular disease did not significantly modify the association between MHR and kidney stones (*P* for interaction>0.05). Intriguingly, we noted a significant interplay between age (20-49/50-79) or gender (female/male) and the MHR-kidney stone linkage (*P* for interaction<0.05). In individuals aged 20 to 49 and among females, there is a stronger correlation between MHR and the risk of kidney stones compared to those aged 50 to 79 and males.

### 3.6 The analysis of threshold effect

**Figure 3:**
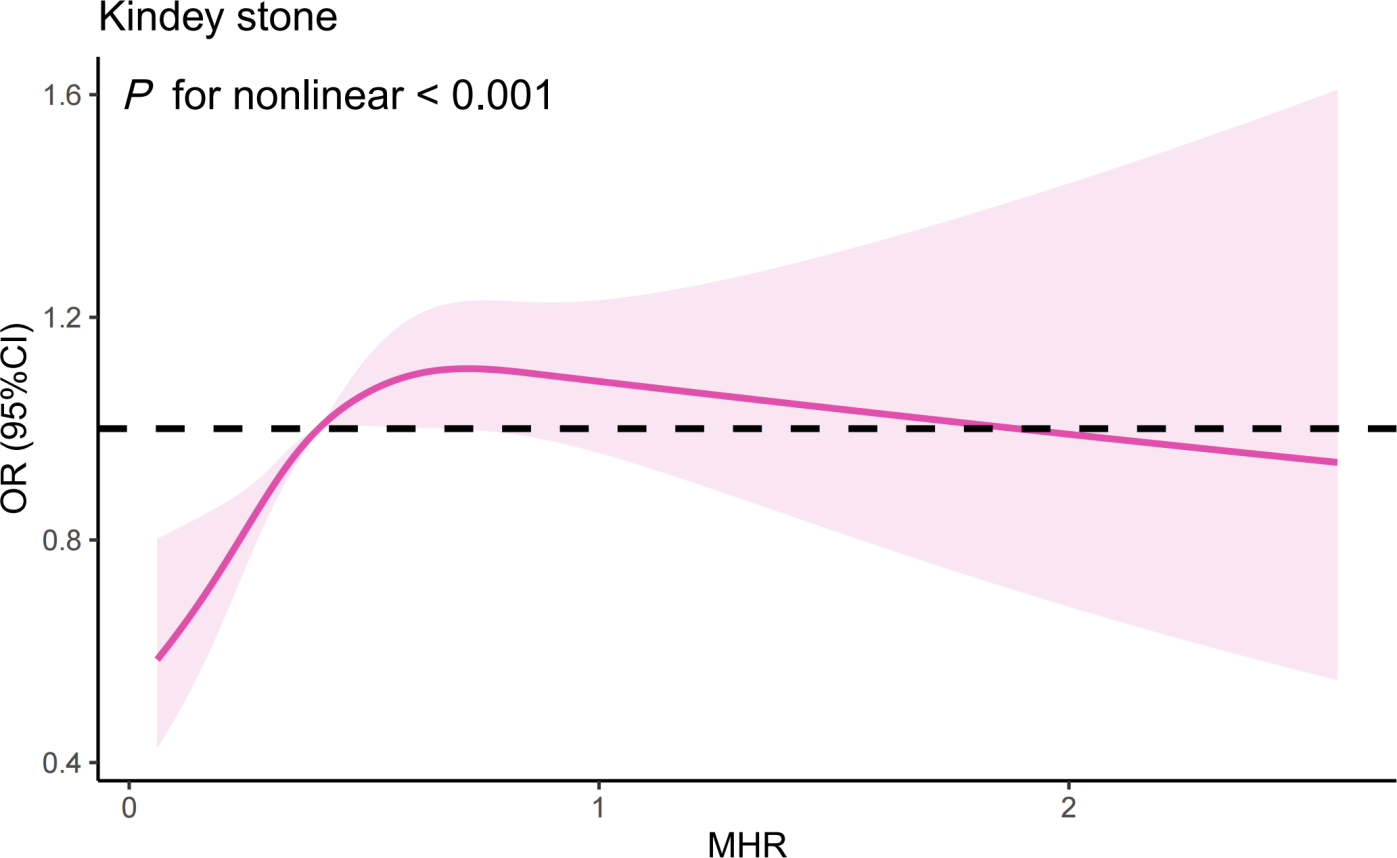
The results of RCS analysis. (Age, gender, and race, annual household income, education level, smokers, hypertension, diabetes, cardiovascular disease, BMI, ALT, AST, GGT, glycohemoglobin, TG, BUN, Scr, and SUA were adjusted).

The RCS analysis suggests a nonlinear relationship between the MHR and the risk of kidney stones in the overall sample (**Figure 3**) (*P* for nonlinear < 0.001). Further investigation using a two-piecewise linear regression model reveals a breakpoint (K) at 0.44 (**Table 5**). To the left of this breakpoint, there is a positive correlation between MHR and the risk of kidney stones, with an OR of 4.57 and a 95%CI ranging from 2.64 to 7.92 (*P*<0.001). To the right of the breakpoint, the association between MHR and kidney stones is not statistically significant, with an OR of 1.02 and a 95% CI from 0.84 to 1.23 (*P*=0.864). There is a significant change across the breakpoint (*P* for logarithmic likelihood ratio<0.001). The results of RCS analysis stratified by age and gender is also provided in **Supplementary Figure 1**.

**Table 5:** Threshold effect analysis of MHR on kidney stone using a two-piecewise linear regression model.

Model	OR (95% CI), *P* value
Fitting by standard linear model	
	1.31 (1.11, 1.54), 0.001
Fitting by two-piecewise linear model	
Breakpoint (K)	0.44
OR1 (<0.44)	4.57 (2.64, 7.92), <0.001
OR2 (>0.44)	1.02 (0.84, 1.23), 0.864
OR2/OR1	0.22 (0.12, 0.42), <0.001
**P** for logarithmic likelihood ratio	<0.001

Adjusted for age, gender, and race, annual household income, education level, smokers, hypertension, diabetes, cardiovascular disease, BMI, ALT, AST, GGT, glycohemoglobin, TG, BUN, Scr, and SUA.

## 4 Discussion

To our knowledge, this is the first population-based study to examine the relationship between MHR and the risk of kidney stones. In the US population, MHR is associated with kidney stones, independently of the effects of other common inflammatory indices (OR=1.22, 95%CI=1.03-1.44, *P*=0.021). RCS analysis indicates a nonlinear relationship, with a saturation threshold of 0.44. Subgroup analysis revealed a stronger correlation between MHR and the risk of kidney stones in individuals aged 20 to 49 and among females. MHR could be a valuable indicator for assessing and predicting the risk of kidney stones.

Inflammation can serve as both a contributing factor in its onset and a consequence of its progression in kidney stone disease (10). In lithogenic environments, an excessive burden of chemical and mineral components, or other sources of inflammatory stimuli, may initially act as triggers, followed by the generation of reactive oxygen species (22). This cascade leads to injury of renal epithelial cells and results in the deposition of calcium oxalate crystals (22). Monocytes and their differentiated counterparts, macrophages, play a crucial role in this context (23, 24). M1-like macrophages facilitate the development of renal calcium oxalate crystals, which is associated with inflammation, fibrosis, and cellular damage within the kidneys (23). In contrast, M2-like macrophages act to suppress the formation of calcium oxalate crystals, thereby potentially protecting against renal pathology (23). On other hand, immune dysfunction in patients with kidney stones can also lead to an excessive generation of reactive oxygen species due to oxalate and calcium oxalate (10). This overproduction of reactive oxygen species can harm the mitochondria of monocytes, compromising their ability to clear stone crystals (10). However, our research indicates that the impact of MHR on kidney stones cannot be fully explained solely from the perspective of immune inflammation and oxidative stress even after adjusting for some representative inflammatory indices. Furthermore, the DCA demonstrates that the evaluation value of the MHR for kidney stones is significantly superior to that of the SII, NLR, and PLR. Therefore, we speculated that the association between MHR and kidney stones extends beyond the impact of inflammatory responses on the formation of kidney stones. Metabolic abnormalities are also intricately linked to the formation of kidney stones (25, 26). Previous research has also revealed a strong connection between MHR and metabolic disorders, such as non-alcoholic fatty liver disease, metabolic syndrome, and polycystic ovary syndrome (27–29). Considering only dyslipidemia, changes in lipid profiles can influence urinary metabolite concentrations and stone composition. Lipid-lowering drugs like atorvastatin also alter urinary citrate and uric acid levels, as well as urine pH (30). Individuals with reduced levels of HDL-c show a significant rise in urinary sodium, oxalate, and uric acid, coupled with a noticeable reduction in urine pH (31). The MHR also serves as an independent marker for cardiovascular diseases (15, 32). Some scholars propose that the build-up of atherosclerotic plaques could result in calcification, which might then breach into the Bellini collecting ducts, thereby heightening the probability of stone formation (33). Thus, we propose that MHR might serve as a comprehensive marker, potentially quantifying the influence of inflammatory responses and metabolic abnormalities on the formation of kidney stones. Further subgroup analyses and interaction tests revealed a more robust correlation between the MHR and kidney stone risk within the demographic of individuals aged 20 to 49 and among females. The inherent independent risk factors of advanced age and male for kidney stone formation could potentially mask the influence of this biomarker on stone risk. Moreover, the divergences in gender and age might reflect distinct pathophysiological pathways in kidney stone genesis across varied populations. The potential confounders present in different gender and age brackets should also be meticulously accounted for in this context.

This research utilized a sample reflective of the ethnic diversity among US adults, yet its limitations must be acknowledged. The cross-sectional nature precludes causal inferences between the MHR and kidney stone risk. Longitudinal studies and clinical trials are essential to verify such associations. Additionally, the exclusion of potential confounders such as metabolic syndrome and nonalcoholic fatty liver disease might have influenced our outcomes. The reliance solely on SII, NLR, and PLR as markers of inflammation on kidney stone could introduce bias. Moreover, as this investigation was based on the US population, its applicability to other populations warrants further exploration.

## 5 Conclusion

In a nationwide study of US adults aged 20-79, a nonlinear relationship was found between MHR and an increased risk of kidney stones. Subgroup analysis indicated this relationship was more pronounced in individuals aged 20 to 49 and among women. MHR could potentially be used as an epidemiological tool to measure the impact of inflammatory responses and metabolic abnormalities on kidney stone formation.

## Data availability statement

The original contributions presented in the study are included in the article/**Supplementary Materials**, further inquiries can be directed to the corresponding author/s.

## Ethics statement

The studies involving humans were approved by the Ethics Review Board of the National Center for Health Statistics. The studies were conducted in accordance with the local legislation and institutional requirements. The participants provided their written informed consent to participate in this study.

## Author contributions

ZW: Writing – original draft, Writing – review & editing. GZ: Writing – original draft, Writing – review & editing. YC: Writing – original draft, Writing – review & editing. TG: Writing – review & editing. QY: Funding acquisition, Writing – review & editing.
