# Supplementary figures and images for: Association between monocyte to high-density lipoprotein cholesterol ratio and kidney stone: insights from NHANES
Source: Front Endocrinol (Lausanne). 2024 Jun 4;15:1374376. doi: 10.3389/fendo.2024.1374376 (PMC11183274; doi:10.3389/fendo.2024.1374376)

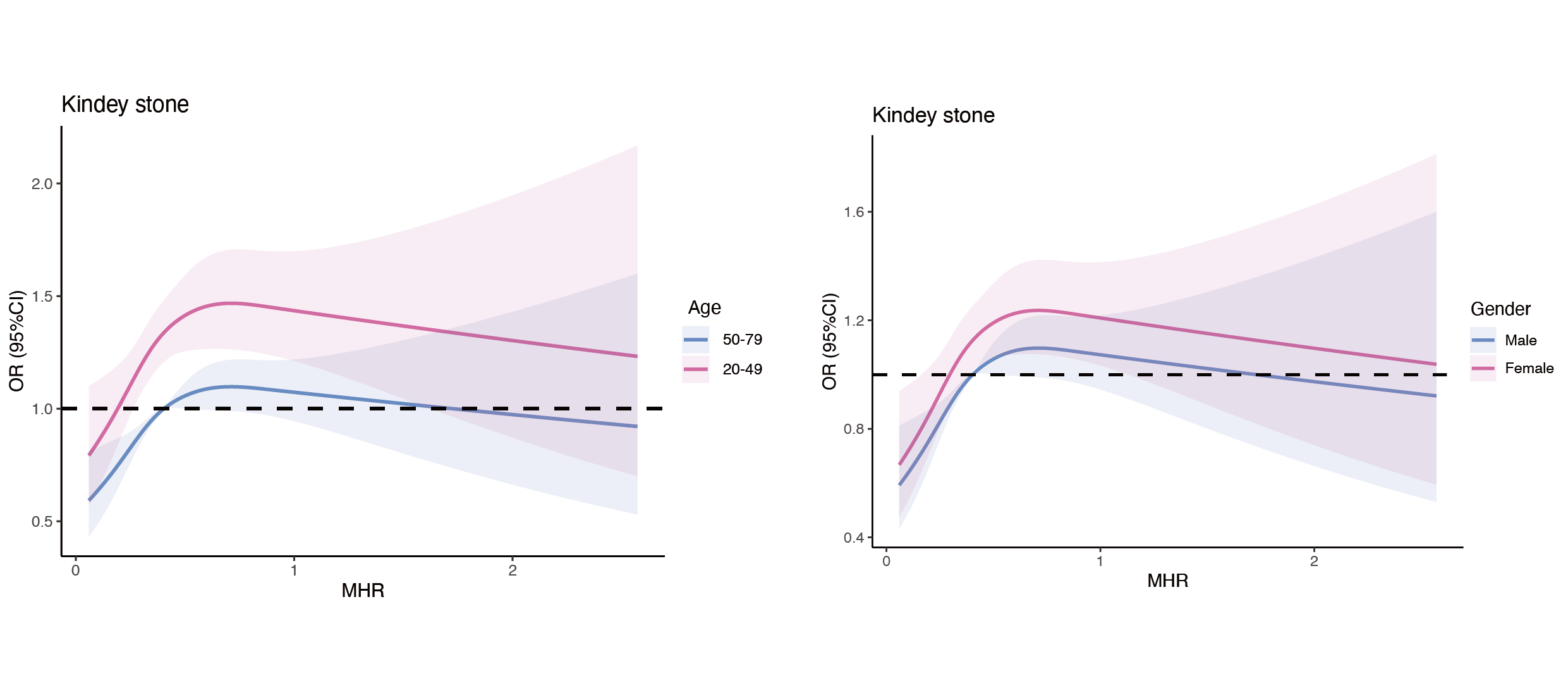

Supplement: Supplementary file 1 [file Image_1.jpeg]
